# Current Hot Potatoes in Atrial Fibrillation Ablation

**DOI:** 10.2174/157340312803760802

**Published:** 2012-11

**Authors:** Laurent Roten, Nicolas Derval, Patrizio Pascale, Daniel Scherr, Yuki Komatsu, Ashok Shah, Khaled Ramoul, Arnaud Denis, Frédéric Sacher, Mélèze Hocini, Michel Haïssaguerre, Pierre Jaïs

**Affiliations:** Hôpital Cardiologique du Haut-Lévêque and the Université Victor Segalen Bordeaux II, Bordeaux, France

**Keywords:** Atrial fibrillation, catheter ablation, complex fractionated atrial electrograms, linear ablation, pulmonary vein isolation.

## Abstract

Atrial fibrillation (AF) ablation has evolved to the treatment of choice for patients with drug-resistant and symptomatic AF. Pulmonary vein isolation at the ostial or antral level usually is sufficient for treatment of true paroxysmal AF. For persistent AF ablation, drivers and perpetuators outside of the pulmonary veins are responsible for AF maintenance and have to be targeted to achieve satisfying arrhythmia-free success rate. Both complex fractionated atrial electrogram (CFAE) ablation and linear ablation are added to pulmonary vein isolation for persistent AF ablation. Nevertheless, ablation failure and necessity of repeat ablations are still frequent, especially after persistent AF ablation. Pulmonary vein reconduction is the main reason for arrhythmia recurrence after paroxysmal and to a lesser extent after persistent AF ablation. Failure of persistent AF ablation mostly is a consequence of inadequate trigger ablation, substrate modification or incompletely ablated or reconducting linear lesions. In this review we will discuss these points responsible for AF recurrence after ablation and review current possibilities on how to overcome these limitations.

## INTRODUCTION 

The description of atrial fibrillation (AF) originating from the pulmonary veins has opened the door for successful catheter ablation of AF [1]. In the last decade, extensive research in the field of AF ablation as well as evolution of techniques and technologies for catheter ablation have advanced the possibilities and success rate for invasive treatment of AF. Nowadays, catheter ablation of AF is the treatment of choice for drug-resistant, symptomatic patients and it is the most frequent ablation procedure performed worldwide [2, 3]. However, some challenges remain in the field of AF ablation, probably being responsible for most ablation failures and the need for repeat procedures in a significant number of patients. In our opinion these challenge are: 1. Permanent pulmonary vein isolation; 2. Understanding of complex fractionated atrial electrograms (CFAE) or what CFAE to target in order to achieve highest arrhythmia-free outcome with least ablation; 3. safe, effective and permanent linear ablation. In the following sections we will discuss these topics. 

## PAROXYSMAL AF ABLATION: PERMANENT PULMONARY VEIN ISOLATION IS THE CHALLENGE 

Pulmonary vein isolation is the cornerstone of AF ablation in both paroxysmal and persistent AF [4]. For successful paroxysmal AF ablation, pulmonary vein isolation is sufficient in most patients [5-7]. Success rates over 70% have been reported after paroxysmal AF ablation and results are maintained during long-term follow-up [5, 8, 9]. Nevertheless, repeat procedures are required in a significant number of cases and recurrent pulmonary vein conduction is responsible for most ablation failures in paroxysmal AF. 

An early study in 43 patients with isolation of superior pulmonary veins during a first procedure and with a repeat procedure in all patients reported rates of pulmonary vein reconduction as high as 79% [10]. After reablation of gap sites, a third procedure in symptomatic patients again revealed repeat reconduction in two thirds of reisolated veins. Nanthakumar *et al*. described reconduction in 42 of 51 previously isolated veins in 15 patients with recurrent symptoms after a first ablation procedure for paroxysmal AF [11]. All patients had 2 or more reconducting veins and in only one patient an extra-pulmonary vein trigger was found. Other groups have confirmed high rates of reconducting veins in patients with ablation failure [8, 12-16]. During repeat ablation procedures in patients with arrhythmia recurrence after pulmonary vein isolation, Gerstenfeld *et al*. showed that triggers awaken by isoproterenol infusion were mainly located in previously isolated but reconducting pulmonary veins [15]. Lim *et al*. reported the rate of reconducting pulmonary veins to be 2.9 during a second procedure, and 2.1 during a third procedure [17]. Two patients undergoing a fourth procedure still had 3 reconducting pulmonary veins. An important study by Verma *et al*. examined pulmonary vein reconduction after pulmonary vein antrum isolation in patients with both paroxysmal and persistent AF [18]. They compared three groups of patients all undergoing a repeat procedure after the index ablation procedure: 1. patients without arrhythmia recurrence; 2. patients maintaining sinus rhythm on antiarrhythmic drugs; 3. patients with arrhythmia despite antiarrhythmic drugs. While in the first group all veins were still isolated in 81% of patients, this rate dropped to 5% and 0% in the other two groups, respectively. Mean number of reconnected veins also increased significantly from group 1 to 3. 

Cheema *et al*. studied the time course of early pulmonary vein reconduction after circumferential pulmonary vein isolation [19]. During a waiting period of 60 minutes after ablation, pulmonary vein recovery was observed in 93% of patients with 50% of pulmonary veins reconnected. Importantly, in one third of reconducting pulmonary veins, conduction recovered during the second 30 minutes of the waiting period. Other studies have confirmed a high rate of early reconduction after pulmonary vein isolation [20]. Finally, even in patients without recurrent arrhythmia after pulmonary vein isolation up to one third of veins may show reconduction, illustrating the fact that some pulmonary veins do not act as a trigger in patients with AF [10, 18]. 

Therefore, almost all patients experiencing recurrent AF after pulmonary vein isolation for paroxysmal AF have at least one reconducting and actively firing pulmonary vein responsible for arrhythmia recurrence. Repeat pulmonary vein isolation in these cases highly increases success rate [5, 13, 14, 18, 21]. To further optimize the success rate of paroxysmal AF ablation without the need of repeat ablations, current limitations of catheter ablation have to be overcome. Technologies, techniques and/or strategies allowing the application of effective and permanent lesions for pulmonary vein isolation without compromising safety are necessary and will be discussed in the next paragraphs. 

### Strategies for Permanent Pulmonary Vein Isolation

The simplest strategy to reduce the rate of pulmonary vein reconduction is to include a waiting period after pulmonary vein isolation and to reablate reconducting pulmonary veins where necessary [19]. After circumferential pulmonary vein antrum isolation Wang *et al*. randomized 90 patients to no further measures, or a waiting period of either 30 or 60 minutes with subsequent reablation in case of reconducting pulmonary veins [22]. They reported about 30% of veins to have recovered, mostly within 30 minutes, and better arrhythmia free outcome in the two groups with a waiting period (61% versus 84% versus 87%, respectively, p=0.04). 

Most groups use entry block into the pulmonary vein assessed by a lasso catheter as a criterion for pulmonary vein isolation. Nevertheless, this does not necessarily imply exit block out of the vein as well. Some groups therefore advocate pacing within the pulmonary veins to document exit block after pulmonary vein isolation in addition to pulmonary vein isolation [23-25]. Because pacing within the pulmonary vein is cumbersome, this strategy has not gained widespread acceptance, and its utility has not been proved in prospective trials. In case of wide circumferential pulmonary vein isolation pacing from within the circumferential ablation line is easier to perform and with this technique complete isolation at the level of the antrum can be obtained. Eitel *et al*. were able to achieve bidirectional block in 95% of patients with this technique and reported a 12 month arrhythmia-free success rate of 84% [26]. 

**Fig. (1):**
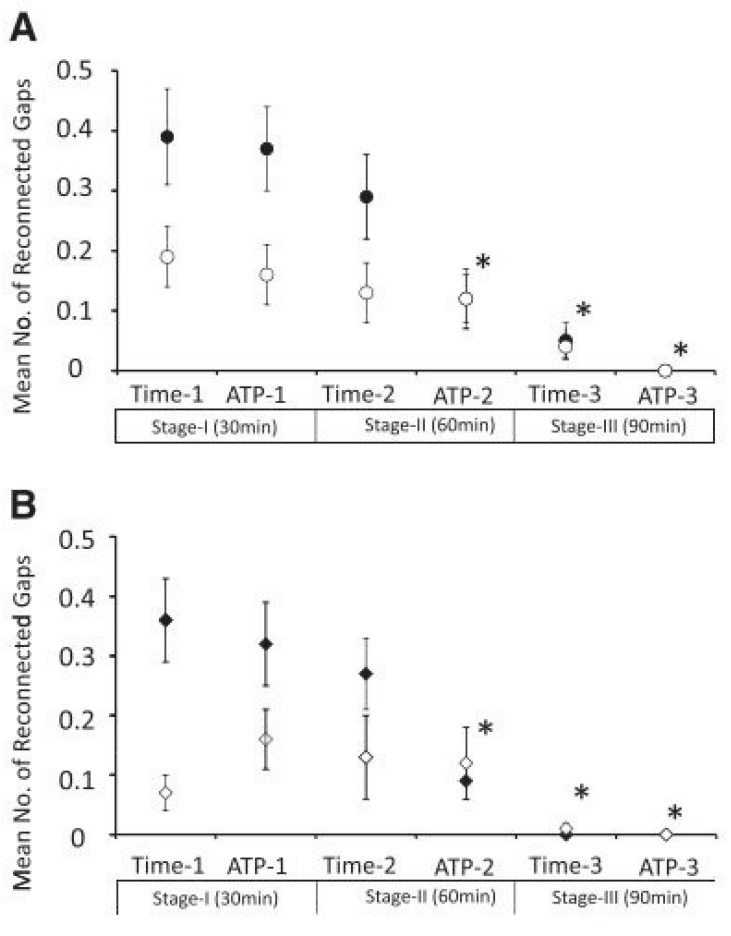
Demonstration of the mean number of reconducting gaps at each provocation step. **A:** right superior (●) and right inferior (○) pulmonary vein. **B:** left superior (♦) and left inferior (◊) pulmonary vein. In both the right and left superior pulmonary veins, the number of reconducting gaps was significantly smaller at ATP 2, time 3, and ATP 3 compared with that of time 1(*p<0.01). Adapted from Yamane *et al*. [35].

In 2004, two groups demonstrated that bolus infusion of adenosine after radiofrequency ablation can reactivate dormant pulmonary vein conduction in 25-35% of isolated pulmonary veins [27, 28]. Three retrospective, single-centre studies tested the hypothesis that the elimination of dormant pulmonary vein conduction by additional radiofrequency ablation improves outcome [29-31]. In all three studies, the arrhythmia-free success rate was significantly higher in the groups with ablation of adenosine-provoked pulmonary vein reconduction after pulmonary vein isolation. In a study by Gula *et al*. adenosine-provoked pulmonary vein conduction had a positive predictive value of 90% and a negative predictive value of 15% for recurrent pulmonary vein conduction during a repeat procedure [32]. Jiang *et al*. compared whether adenosine infusion directly after termination of radiofrequency ablation or a waiting period of 30 minutes will expose a higher number of dormant pulmonary veins [33]. A total of 24% of veins in 64% of patients had reconnected when assessed by either method. Adenosine infusion revealed reconduction in 15% of veins and 30 minutes of waiting in 19%, with moderate agreement between the two methods. Ninomiya *et al*. reported spontaneous, time-provoked reconduction in 25% of pulmonary veins in 62% of patients occurring at a mean of 66 minutes after pulmonary vein isolation [34]. In 10% of reconducting veins it took more than 60 minutes of waiting for reconduction to occur. Subsequent adenosine infusion exposed dormant pulmonary vein conduction in 12% of the remaining veins. Yamane *et al*. most accurately examined time- and adenosine-provoked pulmonary vein reconduction in 75 patients (Fig. **1**) [35]. At steps of 30, 60 and 90 minutes spontaneously occurring pulmonary vein reconductions were ablated, followed by adenosine provocation at each step with ablation in case of dormant pulmonary vein conduction. After 30 minutes, 75 gaps were observed spontaneously in 293 pulmonary veins, and after reablation another 76 gaps were provoked by adenosine infusion, only 5 of whom overlapped with the spontaneously occurring gaps already reablated. After 60 minutes 64 gaps had spontaneously appeared (10 situated in previous gap regions), and adenosine provocation after reablation again revealed 36 gaps (8 situated in previous gap regions). After 90 minutes, only a few gaps were spontaneously observed. In total, time- or adenosine-dependent pulmonary vein reconduction was observed in 81% of patients and in 61% of all pulmonary veins. Most importantly, after one year of follow-up, 92% of patients in this study were free from arrhythmia without antiarrhythmic drugs and after a single procedure. 

Apart from the studies by Artentz and Tritto, all the studies investigating dormant pulmonary vein conduction mentioned above used adenosine injection during continuous isoproterenol infusion [22, 27-30, 33-35]. Datino *et al*. looked at the efficacy of adenosine and isoproterenol to reveal dormant pulmonary vein conduction [36]. In 25 patients dormant conduction was revealed by the combination of adenosine and isoproterenol in 31 veins. Sensitivity to detect dormant pulmonary vein conduction with either adenosine or isoproterenol alone was 87% and 10%, respectively. In a canine model the same group demonstrated that adenosine selectively hyperpolarizes canine pulmonary veins [37]. Pulmonary veins with dormant conduction showed less radiofrequency-induced depolarization than non-dormant veins, allowing adenosine-induced hyperpolarization to restore excitability. As compared to adenosine, isoproterenol also caused hyperpolarization of pulmonary veins but with a smaller magnitude and no cases of isoproterenol-induced reconnection occurred. Therefore, results from basic research suggest that adenosine injection without concurrent isoproterenol infusion is sufficient to reveal dormant pulmonary vein conduction. Whether adenosine testing should routinely be implemented after pulmonary vein isolation remains debatable. Currently, a prospective, multicentre outcome trial is being performed that will help clarify this issue (ADVICE trial) [38]. In this trial, patients with adenosine-provoked dormant pulmonary vein conduction are randomized to further ablation or no further ablation with a follow-up of 12 months for arrhythmia recurrence. 

### Ablation Techniques for Permanent Pulmonary Vein Isolation

Pulmonary vein isolation can be achieved by both segmental pulmonary vein isolation or wide circumferential pulmonary vein isolation [21]. The latter technique requires more extensive, linear ablation, and most operators use electroanatomical mapping systems to guide ablation. Reviews comparing these ablation techniques have found a slightly better arrhythmia-free outcome after wide circumferential pulmonary vein isolation than after segmental pulmonary vein isolation [6, 39]. Importantly, pulmonary vein isolation has to be verified after wide circumferential pulmonary vein ablation, as even with apparently coalescent lesions residual electrical conduction remains in up to 45% of veins [40]. This was corroborated by a randomized study of Khaykin *et al*. in patients with mainly paroxysmal AF undergoing either circumferential pulmonary vein ablation without the endpoint of pulmonary vein isolation or segmental pulmonary vein isolation: patients in the segmental pulmonary vein isolation group had significantly better arrhythmia-free outcome after 2 years (57% versus 27%, p=0.02) [41]. Another interesting study by Liu *et al*. randomized patients undergoing circumferential pulmonary vein ablation to residual gap ablation either at the level of the circumferential line or the pulmonary vein ostium [42]. Freedom from recurrent arrhythmia was significantly lower in patients having ostial residual gap ablation. In patients with paroxysmal AF, high dominant frequency sites and CFAEs are predominantly found within the pulmonary vein antra, pointing to an arrhythmogenic role of these regions in paroxysmal AF [43, 44]. This might explain why circumferential pulmonary vein isolation, when combined with verification of pulmonary vein isolation, is superior to segmental pulmonary vein isolation. 

Another approach for circumferential antral pulmonary vein isolation was chosen by Steven *et al*. [45]. Instead of displaying pulmonary vein potentials to guide pulmonary vein isolation, they continued ablation until pace capture no longer occurred along ablated lines. Using this endpoint, 95% of pulmonary veins were isolated when assessed with a circular catheter. Interestingly, analysis of blinded pulmonary vein electrograms revealed that after isolation was achieved in 50% of pulmonary vein pairs, additional ablation was necessary to achieve loss of pace capture at all ablation sites. Unfortunately, they did notreport arrhythmia-free outcome of patients ablated with this technique. 

### New Technologies for Permanent Pulmonary Vein Isolation 

Table **1** gives an overview of new technologies that are employed for pulmonary vein isolation. The main objective of most of these new technologies is to simplify and shorten the procedure of pulmonary vein isolation. Of course, these new technologies also have to compete with conventional radiofrequency ablation regarding permanent pulmonary vein isolation.

#### Circular Ablation Catheters

Multielectrode catheters have been developed to facilitate pulmonary vein isolation. These catheters have a circular design of variable width and are placed into the antrum of the pulmonary veins. The non-irrigated pulmonary vein ablation catheter (PVAC, Medtronic Ablation Frontiers, Carlsbad, CA) was the first circular ablation catheter developed. With a special generator duty-cycled radiofrequency energy is delivered simultaneously in a unipolar/bipolar mode to all or selected electrodes. In a systematic review of studies using the pulmonary vein ablation catheter, arrhythmia-free success rate was close to 60% after one year in patients with paroxysmal AF [46]. Studies comparing the pulmonary vein ablation catheter with conventional radiofrequency ablation found no difference in arrhythmia-free outcome [47, 48]. In a recent study on 110 patients single procedure arrhythmia-free success rate for paroxysmal AF ablation with the pulmonary vein ablation catheter was 52% [49]. Newer studies suggest that there is a significantly higher rate of silent cerebral ischaemic lesions with the use of the pulmonary vein ablation catheter compared to conventional radiofrequency ablation or cryoballoon ablation [50, 51]. These lesions also tend to be multiple and larger in the pulmonary vein ablation catheter group compared to the other groups. The reason for this higher rate of silent cerebral ischemic lesions is unclear and various explanations have been proposed, of whom the lack of external irrigation is the most obvious explanation. Moderate pulmonary vein narrowing has also been reported in up to 30% of veins ablated by the pulmonary vein ablation catheter [52]. Therefore, the pulmonary vein ablation catheter is not superior to conventional radiofrequency ablation and serious questions regarding its safety have to be addressed in the future. 

Recently, an open-irrigated, circular, multielectrode ablation catheter (nMARQ, Biosense Webster, Diamond Bar, CA) has been developed. A specific generator capable of delivering either uni- or bipolar radiofrequency energy to all or selected electrodes has also been engineered. With this generator, radiofrequency energy delivery can be controlled separately for each electrode. Whether adding irrigation will increase the success rate in paroxysmal AF ablation without increasing the rate of silent cerebral ischemia remains to be proven. Currently, a phase II trial is in the recruiting phase (www.clinicaltrials.gov; NCT01353586) that will answer some of these questions. 

#### Balloon-based Ablation Catheters

Balloon-based ablation systems are another convenient possibility for pulmonary vein isolation and cryoballoon catheters (Arctic Front®, Medtronic CryoCath LP, Quebec, Canada) are the most commonly used. A meta-analysis recently reviewed the results of AF ablation with a cryoballoon catheter [53]. The arrhythmia-free outcome at 1 year was 73% in studies reporting results with a 3-month blanking period after ablation. Two studies comparing cryoballoon ablation with conventional radiofrequency catheter ablation, found no difference in freedom from recurrent AF after 1 year [54, 55]. Details on pulmonary vein conduction during repeat procedures after cryoballoon ablation were reported in 4 studies with an average of 2.7 veins reconducting in 71 patients [53]. Conduction recovery occurred predominantly at inferior sites around ipsilateral pulmonary vein ostia and at the ridge between the left atrial appendage and left-sided pulmonary veins where balloon contact is weakest [56]. Apart from a higher rate of phrenic nerve palsy cryoballoon ablation appears safe. Although not superior to conventional radiofrequency ablation cryoballoon ablation is a viable alternative for pulmonary vein isolation. 

Different kinds of “hot” balloons have been developed using ultrasound, laser or thermal energy for lesion formation. A balloon-based high-intensity focused ultrasound catheter (ProRhythm, Ronkonkoma, NY) was able to achieve acute pulmonary vein isolation in 89% of veins [57]. Because of a high complication rate, including fatal outcome, clinical use was halted [58]. Of the initially treated patients, 79% were free from recurrent arrhythmia after 2 years without antiarrhythmic drugs [59]. 

A thermal balloon catheter of 25-35 mm diameter filled with saline and contrast medium that can be heated by radiofrequency energy delivered *via* a coil electrode within the balloon (Toray Industries, Urayasu, Chiba, Japan) has been tested in two small studies [60, 61]. After isolation of all pulmonary veins and creating a posterior box lesion, 95% of 63 patients with paroxysmal AF were free from recurrent arrhythmias after 11 months. This promising technology needs further studies to prove its safety and superiority over conventional radiofrequency ablation. 

Since 2009, several studies using a balloon-based, visually-guided laser ablation catheter (Endoscopic Ablation System, CardioFocus, Marlborough, MA) for pulmonary vein isolation have been published. Using this technology, the operator sees the laser beam and can ensure overlap of consecutive energy deliveries but the lesion itself is not visible. Close to 100% of pulmonary veins can be isolated with this catheter and after 3 months up to 86% remain isolated [62]. Single-procedure, arrhythmia-free outcome after 12 months without antiarrhythmic drugs is reported at 60% [63, 64]. Some concerns have been raised as more severe oesophageal thermal lesions were found using the laser-balloon catheter compared to conventional radiofrequency ablation [65]. Additional data on safety and efficacy of the laser-balloon catheter are needed before widespread clinical use can be recommended. 

Apart from the more compliant thermal and laser balloons, all balloon-based ablation systems share the characteristic to isolate pulmonary veins at the level of the pulmonary vein ostium, sparing the antrum [66-68]. As discussed above, ostial rather than antral pulmonary vein isolation may result in a lower arrhythmia-free success rate in paroxysmal AF ablation and this may be a disadvantage of noncompliant balloon-based ablation systems. These systems also require one to push on the balloon to maximize contact and allow for complete interruption of pulmonary vein flow. 

#### Remote Navigation

Remote navigation systems allowing for better catheter manoeuvrability and stability have been introduced into electrophysiology labs. Currently, two different systems, the Niobe^®^ magnetic navigation system (Stereotaxis, St. Louis, MO) and the Sensei® robotic navigation system (Hansen Medical, Mountain View, CA) are commercially available. Both systems are equipped with conventional radiofrequency ablation catheters. Using the remote magnetic navigation system, arrhythmia-free success rate was not different to conventional, manual catheter ablation in different studies and the use of this system appears safe [69-73]. Because the remote magnetic navigation system is time- and resource-consuming and has not proved superior to conventional pulmonary vein isolation it has not gained widespread acceptance for pulmonary vein isolation. 

With robotic remote navigation, pulmonary vein isolation is also feasible [74]. Because contact force is improved by the use of a special sheath, tactile feedback has been implemented into the system to lower the risk of cardiac perforation and oesophageal lesions [74, 75]. Compared to conventional, manual radiofrequency ablation, the use of remote robotic navigation for pulmonary vein isolation yields similar arrhythmia-free success rate [76-78]. In patients remapped 3 months after catheter ablation by robotic navigation irrespective of outcome, 43% of all pulmonary veins had reconnected [79]. Restudied patients without AF recurrence had significantly fewer recovered pulmonary veins. 

#### Pressure Control

**Fig. (2):**
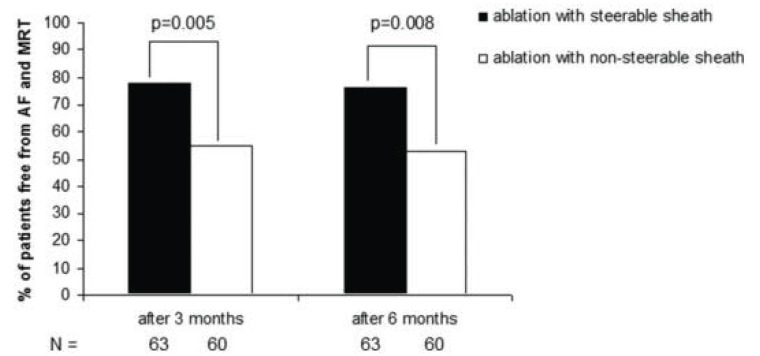
Bar graphs showing single procedure success (freedom from arrhythmia) 3 and 6 months after AF ablation using a steerable sheath versus a non-steerable sheath. MRT= Macro Reentrant Tachycardia Adapted from Piorkowski *et al*. [82].

Both a case-control study by Piorkowski and a retrospective study by Arya reported a very high single procedure success rate using a steerable sheath for circumferential pulmonary vein ablation, notably with pulmonary vein isolation in only 39% of patients in the retrospective analysis [80, 81]. In a third study by the same group randomizing patients to ablation with a steerable or non-steerable sheath, single procedure success rate after 6 months was significantly higher in the steerable sheath group (53% versus 76%, p=0.008, (Fig. **2**)) [82]. The use of a steerable sheath could therefore allow for more permanent lesions and translate into better arrhythmia-free outcome. However, another group randomizing patients to AF ablation with a steerable or non-steerable sheath found no difference in arrhythmia-free outcome between the two groups [83]. Nevertheless, it is plausible that the use of a steerable sheath allows for better pressure control resulting in more uniform lesion formation and permanent pulmonary vein isolation, and that this simple and available technology is probably underused. Importantly, complication rates in the studies comparing outcome of pulmonary vein isolation with the help of a steerable sheath versus a non-steerable sheath were not different [81-83]. 

Catheter manufacturing companies have recently developed catheters capable of measuring and displaying contact force at the catheter tip. Animal studies with these catheters confirmed a linear correlation between contact force and lesion size [84, 85]. Catheter ablation of AF using contact force technology showed high interoperator variability of mean contact force at ablation sites [86]. One study reported a significantly lower contact force during ablation at sites with acute pulmonary vein reconduction compared to sites without acute pulmonary vein reconduction [87]. Whether catheter ablation of AF with force feedback will result in better lesion control and higher success rate is currently being investigated in different prospective studies (www.clinicaltrials.gov; NCT01278953 and NCT01385202). Both the use of a steerable sheath and force feedback aim at optimizing pressure control and might be even more advantageous when used in combination.

#### Others

A novel endoscopic mapping and ablation catheter (IRIS Ablation Catheter, Voyage Medical, Campbell, CA) that allows direct visualization of the endocardial surface combined with irrigated radiofrequency ablation is being developed and used in research protocols. With this catheter it is possible to see lesion formation during ablation even in the blood-filled vascular system. Residual gaps at ablation sites can be visualized with this technology and this might transform into better lesion control and contiguous ablation. Results of human studies have to be awaited. 

For some years, pulmonary vein isolation has been performed by a growing number of surgeons with a minimally invasive, video-assisted thoracoscopic technique [88]. Studies have reported variable success rates depending on the length of follow-up, methods for detection of recurrent AF and characteristics of patients included [89-93]. One study randomized AF patients to either minimally invasive surgical or conventional catheter ablation [90]. Importantly, the majority of patients in this study had failed a previous conventional catheter ablation. Freedom from recurrent AF after 12 months of follow-up was significantly higher after minimally invasive surgical ablation, but so was the procedural adverse event rate. Minimally invasive epicardial ablation has to find its place in the treatment of AF. However, in light of the higher adverse event rate and invasiveness of the procedure, stand alone surgery is unlikely to play an important role in paroxysmal AF ablation in near future. 

## SUMMARY

Pulmonary vein isolation is a very effective treatment for paroxysmal AF. Inability to achieve permanent pulmonary vein isolation is responsible for recurrent AF and the need for repeat procedures. Wide circumferential pulmonary vein isolation with confirmation of exit block instead of segmental pulmonary vein isolation and repeat pulmonary vein assessment for early pulmonary vein reconduction 30-60 minutes after isolation with reablation as necessary may support permanent pulmonary vein isolation. Whether additional ablation of adenosine-provoked pulmonary vein reconduction after isolation results in higher arrhythmia-free outcome is currently being investigated in a multicentre, randomized study. So far, balloon-based ablation systems and non-irrigated, circular, multielectrode ablation catheters, as well as remote navigation, have failed to outperform conventional radiofrequency ablation. Study results of an irrigated, circular, multielectrode ablation catheter are awaited and first clinical experience with a thermal balloon catheter is encouraging. The integration of force feedback into catheter design and the use of steerable sheaths are promising and may allow for better lesion control and permanent pulmonary vein isolation. Minimally-invasive surgical pulmonary vein isolation will have to prove its superiority over conventional catheter ablation in larger, randomized trials to be considered as a first-line option for paroxysmal AF ablation. 

## PERSISTENT AF ABLATION: CFAEs AND LINES ARE THE CHALLENGE 

The Cox-maze procedure introduced by Dr. James Cox in 1987 was the first successful invasive treatment for AF [94]. Several modifications of the original procedure resulted in the classical, cut and sew Cox-maze III procedure and, replacing the incisions by ablation lines, the Cox-maze IV procedure [95]. The concept of the Cox-maze surgery was to interrupt the multiple wavelets of AF and to direct atrial activation through a maze-like system involving both atria. During the procedure all pulmonary veins are isolated by a box lesion. High rates of arrhythmia-free outcome have been reported after the Cox-maze III and IV procedures, although procedure technique, type of AF and type of follow-up are heterogeneous among different studies and outcome tends to be better after the cut and sew Cox-maze procedure [96-99]. But most importantly, the success of the Cox-maze procedure proved that atrial compartmentalization by a set of linear lesions can effectively suppress AF. In an attempt to reproduce the success of the surgical maze procedure, cardiac electrophysiologists started to imitate the Cox-maze procedure by ablating similar sets of linear lesions in the right atrium or both atria [100, 101]. However, success rate of endocardial linear ablation was only modest, probably because the endpoint was not complete block of the ablated lines. The discovery of AF originating from pulmonary vein triggers shifted interest towards isolation of the pulmonary veins [1]. However, it soon became apparent that exclusive pulmonary vein (antrum) isolation has a low success rate in persistent AF [17, 102, 103]. Therefore, the focus turned back to substrate modification for persistent AF ablation in addition to pulmonary vein isolation. 

**Fig. (3):**
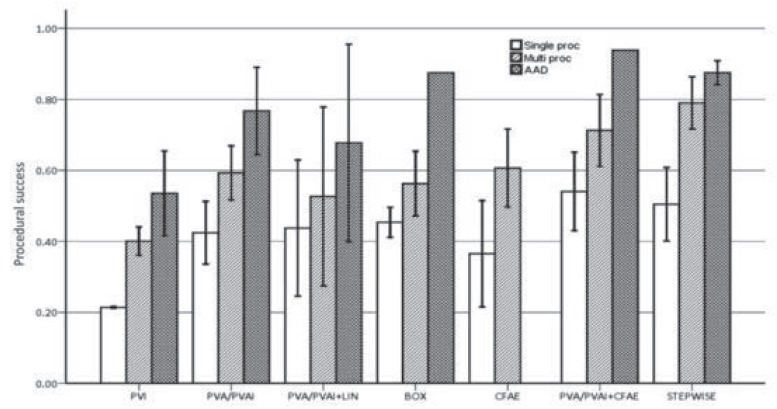
Clinical success of various ablation techniques for persistent/long-standing persistent atrial fibrillation. Rates are shown for single-procedure, drug-free success (white), multiple-procedure success (diagonal cross hatch), and anti- arrhythmic drug (AAD)-assisted success (dark double hatch). BOX: posterior wall isolation; LIN: conventional linear ablation; PVA: pulmonary vein antrum ablation; PVAI: pulmonary vein antrum isolation; PVI: pulmonary vein isolation; STEPWISE: stepwise ablation technique. Adapted from Brooks *et al*. [115].

Parkash *et al* did a meta-analysis including only randomized controlled trials [6]. They found four studies randomizing patients with persistent AF to pulmonary vein isolation or pulmonary vein antrum ablation with or without the addition of linear ablation. Single procedure outcome was in favour of additional linear ablation. Nademanee was the first to describe a technique of CFAE ablation alone in patients with paroxysmal and persistent AF and reported a high rate of arrhythmia-free outcome after one year [104]. However, other groups were not able to replicate this success rate with CFAE ablation alone [105-108]. Since, several studies investigated the incremental benefit of CFAE ablation when added to pulmonary vein isolation. In patients with persistent AF, most studies, as well as a meta-analysis, are in favour of a combined approach of pulmonary vein isolation and CFAE ablation [107, 109-114]. Subsequently, different ablation strategies for persistent AF ablation were developed. After pulmonary vein (antrum) isolation, CFAE ablation or linear ablations were added to the ablation strategy either separately or combined. Brooks *et al*. published a detailed review of reported outcomes of different ablation strategies for long-standing persistent AF (Fig. **3**) [115]. They found very low success rate after segmental pulmonary vein isolation and slightly higher success rate after pulmonary vein antrum ablation with or without pulmonary vein isolation. The addition of either CFAE ablation or linear ablation to pulmonary vein antrum ablation both increased success rate to a similar extend. In another study, Estner *et al*. randomized 116 patients with persistent AF to either circumferential pulmonary vein isolation plus additional lines, or CFAE ablation plus ostial pulmonary vein isolation [116]. Arrhythmia-free, single procedure outcome after 12 months was not different among the two groups (37% versus 39%), neither was multiple procedure outcome (54% versus 56%). Therefore, both additional CFAE ablation and linear ablation after pulmonary vein isolation increase the arrhythmia-free outcome in persistent AF. In the above mentioned review by Brooks *et al*, the best antiarrhythmic drug-free outcome after multiple procedures in persistent AF was observed in studies including both CFAE and linear ablation, as performed in the stepwise ablation approach (Fig. **3**) [115]. 

The stepwise AF ablation approach was first introduced by the Bordeaux group in 2005 [117]. Sequential pulmonary vein isolation, isolation of the great thoracic veins and ablation of CFAE sites were performed in a random, stepwise order in 60 persistent AF patients, with subsequent linear ablation in case of persisting AF or macroreentrant atrial tachycardia. With this approach, AF was terminated to either sinus rhythm or atrial tachycardia in 87% of patients. In more than half of the cases, AF terminated after the first three steps (venous isolation and CFAE ablation). In the remaining patients linear ablation was required and ultimately resulted in termination of AF in another third of patients. Of 87 atrial tachycardias observed after AF termination, 49 were macroreentrant atrial tachycardias necessitating linear ablation at the left atrial roof, mitral isthmus, or cavotricuspid isthmus. With this stepwise ablation approach, and after repeat ablation procedures as necessary, the arrhythmia-free outcome after 11 months was 95% [118]. Another group recently confirmed a high success rate after persistent AF ablation using the stepwise ablation approach [119]. While single procedure arrhythmia-free outcome after 27 months was only 27% in this study, multiple procedures outcome at 15 months after a mean of 2.3 procedures was 79%. The strongest predictor of both single and multiple procedure outcomes was AF termination during the index procedure. Both during the index, as well as repeat procedures, a high rate of macroreentrant atrial tachycardias occurred, necessitating linear ablation. 

When linear ablation is not part of the ablation protocol, the AF termination rate during persistent AF ablation is lower. After pulmonary vein antrum isolation and CFAE ablation but no linear ablations Elayi *et al*. reported AF termination in 58% of 306 persistent AF patients [120]. In that study, AF termination did not predict arrhythmia-free outcome. However, with the addition of linear ablation as in the full stepwise ablation approach a higher rate of AF termination can be achieved and in these patients AF termination during the index procedure has impact on long-term follow-up. This was demonstrated in a study by O’Neill *et al*. in which termination of persistent AF was achieved in 85% of patients during the stepwise ablation approach [121]. Among patients with AF termination, this occurred during CFAE ablation in 68% and during linear ablation in 21%. After repeat ablation, sinus rhythm was maintained in 95% of patients in whom AF was terminated during the index procedure compared with 52% in those in whom AF could not be terminated. Knecht *et al*. analyzed the necessity of linear ablation in 154 out of 180 persistent AF patients, in whom AF terminated during the stepwise ablation approach [122]. Ablation of the roof line was necessary in 95% of patients and ablation of the mitral isthmus line in 77% of patients, either for AF termination or because of subsequent macroreentrant tachycardia involving the roof or mitral isthmus. The benefit of mitral isthmus ablation has been questioned by some groups. One study randomized patients with mitral isthmus dependent atrial tachycardia after persistent AF ablation to either cardioversion with repeat pulmonary vein isolation and non-pulmonary vein trigger ablation or ablation of the mitral isthmus [123]. Not surprisingly, arrhythmia recurrence was higher in the group without repeat pulmonary vein isolation. 

In conclusion, both CFAE ablation and linear lesions are necessary in persistent AF ablation to achieve a high rate of AF termination during the index procedure. Termination of AF during ablation is predictive of a favourable outcome and should be the goal of the stepwise ablation approach, with additional ablation targeting subsequent atrial tachycardias as necessary. 

## PERSISTENT AF ABLATION, CHALLENGE ONE: CFAEs

### CFAE Definition

In his original study, Nademanee defined CFAEs as: 1) atrial electrograms that have fractionated electrograms composed of two deflections or more, and/or perturbation of the baseline with continuous deflection of a prolonged activation complex over a 10 second recording period; 2) atrial electrograms with a very short cycle length (≤120 ms) averaged over a 10 second recording period [104]. By this definition, CFAE are either multicomponent electrograms (time domain analysis) and/or discrete high frequency electrograms (frequency domain analysis)[104,105]. However, classifying an electrogram as complex fractionated is subjective with substantial interobserver variability. Furthermore, the original CFAE definition of Nademanee has evolved over the years. For example, the number of deflections required for an electrogram to be fractionated has been augmented to 3 or higher, or the minimal duration of continuous electrical activity more precisely defined (e.g. ≥50 ms) [124, 125]. Furthermore, not all studies include an averaged cycle length ≤120 ms as a CFAE criterion. This is not without consequences, as there is poor anatomic overlap between CFAE sites defined by multicomponent electrograms and CFAE sites defined by averaged cycle length ≤120 ms [126]. Software with automated algorithms for CFAE detection has been developed and integrated into 3D mapping systems (Ensite NavX, St Jude Medical, Minneapolis, MN, USA; Carto 3, Biosense Webster, Diamond Bar, CA, USA). Diagnostic accuracy of these CFAE detection algorithms as compared to visual CFAE detection is highly dependent on parameter settings and visual CFAE definition [127-129]. Depending on the software used and parameter settings applied, CFAEs can be found in over 80% of analyzed sites in patients with paroxysmal and persistent AF, questioning specificity of CFAE definitions [130]. Because no consensus exists on how to define CFAEs and different techniques are applied for automatic electrogram analysis, studies are difficult to interpret and to compare. 

### Basis of CFAE Formation 

With bipolar recording, which is preferentially used for AF ablation by most centres, the potential generated is the difference of the potentials recorded by the two closely spaced electrodes [131]. The major advantage over unipolar recording is that far-field signals are subtracted out. The “field of view” of bipolar recording comprises the tissue underlying the two electrodes and all signals generated by activation underneath the electrodes are recorded, but still spanning an area of several millimetres. Most CFAEs are in fact real signals from the nearer field of the two electrodes and only rarely are true artifacts. 

**Fig. (4):**
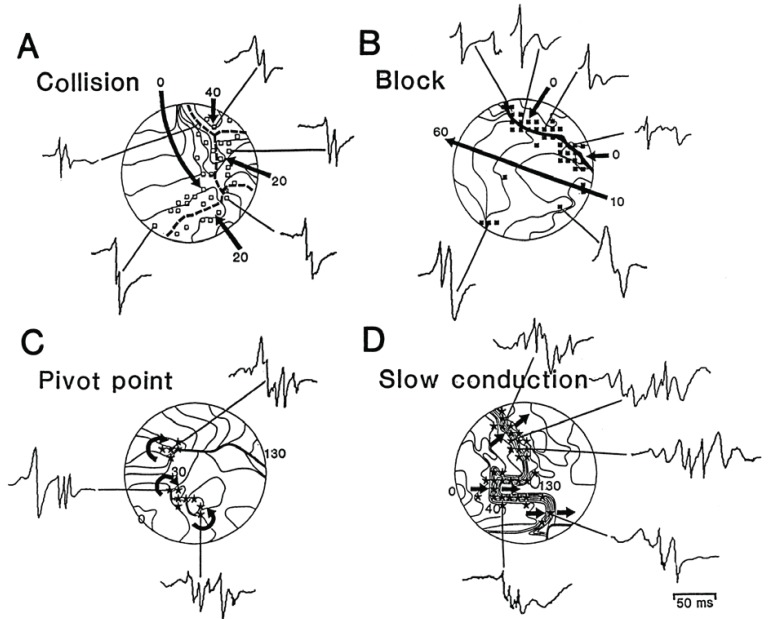
Comparison of the electrogram morphology and the underlying pattern of activation as recorded during AF. **A:** Four wave fronts (arrows) invade the mapping area from different directions and collide along the dashed line. Along either side of this line of collision, short double-potentials were recorded (□). **B:** Long-double electrograms (▪) are shown to be recorded at a line of functional conduction block (thick line). Fragmented electrograms (*) were recorded both at pivot points (**C**) and from areas with slow conduction (**D;** crowding of isochrones). Isochrones were drawn at 10-ms interval. Arrows indicate the direction of activation. Numbers indicate activation times in milliseconds. Adapted from Konings *et al*. [134].

A multitude of mechanisms have been proposed to be responsible for CFAE formation [132]. Remote activation of adjacent structures and local, atrial structural complexity are mostly responsible for the formation of multicomponent atrial electrograms (according to the time domain definition of CFAEs). The human atria are complex anatomical structures with anatomical and electrical tissue heterogeneity (tissue anisotropy) at multiple sites. The orifices of the pulmonary veins and caval veins, the coronary sinus, the interatrial septum and the pectinate muscles of both atrial appendages all contribute to this complexity. Furthermore, throughout both atria there is a complex 3D muscular myoarchitecture with different layers blending one into each other [133]. Konings *et al*. elegantly demonstrated that during AF zones of slow conduction and pivot points at the end of lines of functional block can develop and result in fractionation of the unipolar atrial electrogram (Fig. **4**) [134]. These zones were absent in sinus rhythm and no sites of preferential conduction block or slow conduction existed in AF. With detailed epicardial mapping of left and right human atria in patients with either induced or persistent AF Allessie *et al*. found many narrow wavelets propagating simultaneously through the atrial walls [135]. The lateral boundaries of these wavelets were formed by lines of functional conduction block, predominantly oriented parallel to the atrial musculature. Lines of block were not fixed but continuously changed. In another study, Berenfeld *et al*. paced sheep right atria at increasingly higher frequencies [136]. With pacing above a “breakdown frequency” of 6.5 Hertz right atrial activation changed from periodic to completely variable from beat to beat and transformed into fibrillatory conduction. Such frequency-dependent changes of activation pattern correlated well with branch sites of the pectinate musculature in the right atrium. In the study of Konings *et al*. the rate of fragmented potentials also increased with shortening of AF cycle length [134]. High density activation mapping has confirmed such dynamic changes of fractionated atrial electrograms during AF in humans, with CFAEs developing mainly after regional shortening of AF cycle length [124]. In a recent study Lalani *et al*. used basket catheters to measure biatrial conduction time during superior pulmonary vein pacing at accelerating cycle lengths [137]. In most cases, acceleration-dependent slowing of atrial conduction (conduction velocity restitution) preceded AF initiation and in patients with persistent AF, this was accompanied by an abrupt vector shift in atrial activation, indicating conduction block as responsible mechanism. Thus, shortening of AF cycle length results in slow conduction and functional conduction block at sites of tissue anisotropy. This leads to wave front collisions and wave breaks at anatomic barriers or at pivot points at the end of lines of functional block und ultimately to fibrillatory conduction. The finding that cycle length shortening at sites of tissue heterogeneity is responsible for CFAE formation is corroborated by the fact that sites displaying CFAEs during AF usually have normal voltage in sinus rhythm, suggesting absence of structural scar [138]. In fact, regions of CFAE during AF only marginally overlap with regions of fractionation during sinus rhythm or coronary sinus pacing [138, 139]. On the other hand, sinus rhythm fractionation mainly occurs at sites of wave-front collisions which are similarly distributed in patients with and without AF, and these sites can be varied by coronary sinus pacing. Temporal and spatial stability of CFAE sites during AF is reported to be high [140, 141]. This can be explained by the fact that CFAEs mostly occur at sites of tissue heterogeneity, where cycle length shortening will repeatedly lead to CFAE formation. To clarify the mechanism of CFAE formation, Narayan *et al*. recorded monophasic action potentials at CFAE sites mapped with a multipolar catheter [142]. According to the monophasic action potentials the mechanism responsible for CFAE formation in two thirds of CFAE sites was remote activation superimposed on bipolar AF electrograms. This CFAE mechanism was most prevalent at anatomic junctures such as the interatrial septum and coronary sinus. Both the septum and coronary sinus are well known locations of high CFAE prevalence [104, 117]. Supporting this finding, most CFAEs are found in the proximal part of the coronary sinus, where the muscular sleeve is most prominent with a complex 3D anatomy[143]. The stereotypical distribution of multicomponent CFAEs to anatomic areas of tissue heterogeneity or anatomical junctures, the absence of scar in sinus rhythm and the relationship to preceding AF cycle length shortening suggest that multicomponent CFAEs are a passive phenomenon and not sites of a focal source. 

As mentioned above, the CFAE definition involves not only multicomponent atrial electrograms but also electrograms with very short cycle length (according to the frequency domain definition of CFAEs). While the cycle length is easy to measure at sites displaying distinct electrograms, this is less feasible at sites with multicomponent atrial electrograms. To overcome this limitation, spectral analysis of intracardiac recordings using fast Fourier transformation has been implemented [144, 145]. With spectral analysis, local dominant frequency of activation is represented by the largest peak in the fast Fourier transform [144, 146]. Prashanthan *et al*. constructed 3D dominant frequency maps in patients with paroxysmal and persistent AF [147]. High dominant frequency sites, defined as sites surrounded by atrial tissue with a decreasing frequency gradient ≥20%, were mainly located within the pulmonary veins in paroxysmal AF and throughout both atria in persistent AF. Mandapati *et al*. used a combination of optical and bipolar electrode recordings to find sites of periodic activity during AF and to identify their mechanism in isolated sheep hearts with pacing and acetylcholine induced AF [148]. The highest dominant frequency sites were mainly localized to the posterior left atrium. They were able to demonstrate that such sources corresponded to vortex-like reentry around minuscule cores, i.e. being a functional reentry (rotor). Using the same model, Kalifa *et al*. showed that the outer limit of the highest dominant frequency site is the area where most fractionation occurs [149]. Stiles confirmed a close spatial relationship between fractionated electrograms and high dominant frequency sites in patients with AF [150]. Eighty percent of high dominant frequency clusters were within 10 mm of fractionated electrograms in their study. Lee *et al*. also found a poor anatomic overlap of epicardial multicomponent CFAEs with CFAE sites defined by short cycle length [126]. Multicomponent CFAEs occurred adjacent to and surrounded areas of high dominant frequency, consistent with the hypothesis that multicomponent CFAEs represent areas of wavebreak around a high-frequency focus. In the above mentioned study by Narayan *et al*. 8% of CFAE sites demonstrated discrete rapid monophasic action potentials and pansystolic local activation with adjacent activation spanning complete AF cycle length [142]. Sites with this CFAE pattern exhibited high narrow dominant frequency peaks, rate stepdown to surrounding sites and were only observed at the pulmonary vein antra and posterior left atrial wall, features consistent with functional reentry as described in sheep models of AF [149]. Atienza *et al*. mapped the posterior left atrial wall of AF patients with a spiral catheter and used a computer simulation of rotors to interpret the results. They also found that electrogram fractionation at the posterior wall results from fibrillatory conduction and are a consequence of the dynamic interaction between high-frequency reentrant sources and the atrial anatomy. In their computer model, cycle length shortening may be the consequence of a drifting functional reentry (doppler effect). However, because of limitations in spatial resolution of current mapping systems the exact mechanism of such high dominant frequency sites has not been clarified and remains a matter of debate. Both a focal automatic or triggered source, as well as functional or anatomical reentry, are possible explanations. Whatever the mechanism, these high dominant frequency sites are probably important drivers for maintaining AF. Nevertheless, in the above mentioned study by Allessie *et al*. no evidence for the presence of stable foci or rotors was found in several thousand epicardial maps of human atria [135]. 

Another component that may affect CFAE formation is the autonomic nervous system. In a canine model, CFAE prevalence was increased by topical application of acetylcholine onto the right atrium and decreased by ablation of ganglionated plexi [151]. Both the prevalence of CFAEs and dominant frequency decreased with distance from ganglionated plexi [152]. In a clinical study combined muscarinic and beta-adrenergic blockade significantly reduced the prevalence of CFAEs independent of AF cycle length in patients with AF ongoing for more than 24h and prevented time-dependent rise in CFAE prevalence in the early phase of AF [153]. However, another study found a significant reduction of CFAE prevalence upon autonomic blockade in paroxysmal but not in persistent AF patients [154]. In that study, reduction of CFAE prevalence correlated with an increase of AF cycle length by autonomic blockade. In the left atrium CFAEs can often be found at presumed anatomic sites of ganglionated plexi which are located close to the pulmonary vein antrum [155]. 

### Why Does CFAE Ablation Work?

From a mechanistic point of view, optimal target sites for ablation are sites harbouring drivers or perpetuators of AF. These would be sites with an active focus (because of enhanced automaticity or triggered activity), functional reentry (rotor), or sites that are part of an anatomical micro- or macroreentry. As discussed above, addition of CFAE ablation to pulmonary vein isolation translates into a more favourable outcome. During the stepwise ablation approach AF cycle lengths in the left and right atrial appendages are monitored. Normally, catheter ablation of CFAEs results in gradual prolongation of AF cycle length and ultimately in termination of AF to either atrial tachycardia or sinus rhythm in up to 80% of patients [117, 119, 156]. An increase of AF cycle length during ablation of a specific CFAE site therefore is a strong argument for an important contribution of that site to AF maintenance. During the stepwise ablation approach for persistent AF, greatest magnitude in AF cycle length prolongation generally is observed during ablation at the anterior left atrium, coronary sinus and the junction of the pulmonary veins with the left atrium [117]. 

**Fig. (5):**
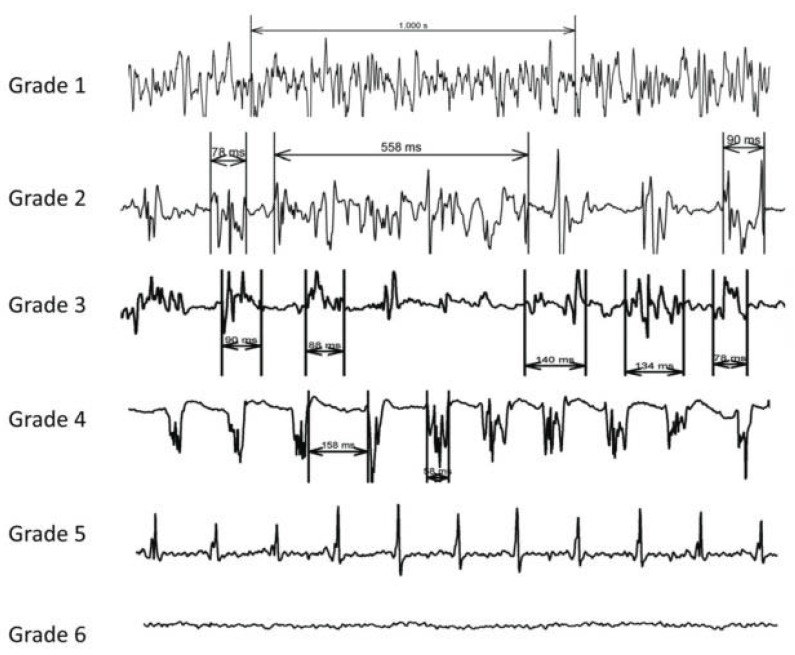
Classification of CFAE from most complex CFAE (**grade 1**) to normal electrogram (**grade 5**) in the study by Hunter *et al*. Grade 6 corresponds to scar tissue. Adapted from Hunter *et al*. [158].

Takahashi *et al*. looked at characteristics of atrial electrograms predictive of slowing or termination of AF during stepwise ablation of persistent AF [157]. A higher percentage of continuous electrical activity or the presence of a temporal gradient of activation were predictive of favourable ablation sites, whereas bipolar voltage, dominant frequency, fractionation index and local cycle length were not. In a recent study Hunter *et al*. demonstrated that AF cycle length prolongation was more likely when ablating more fractionated atrial electrograms (Fig. **5**) [158]. In this study, overall rate of AF cycle length prolongation was the same regardless of whether more or less fractionated atrial electrograms were ablated first, but ablation of more fractionated atrial electrogram first reduced the number of remaining less fractionated atrial electrograms. 

During left atrial ablation a divergent pattern of AF cycle length prolongation as measured in the left and right atrial appendage can be observed in about 20% of persistent AF patients, suggesting that the right atrium is the driver of AF [156]. In these patients, termination of AF can be achieved with additional right atrial ablation. This clearly shows that drivers and/or substrate necessary for AF perpetuation are not confined to the left atrium but also involve the right atrium in some cases. 

In only a few studies high dominant frequency sites were targeted during ablation, and most studies involved only a limited number of patients with persistent AF [147, 159, 160]. In these studies, ablation of high dominant frequency sites in patients with persistent AF had no major impact on AF cycle length and did not result in a high rate of AF termination, but the number of patients was low. So far, no study has compared the arrhythmia-free outcome after ablation of either high dominant frequency sites or specific CFAE sites versus standard CFAE ablation. 

Ganglionated plexi are located in the epicardial fat pads and ligament of Marshall and therefore are less accessible to endocardial radiofrequency ablation [161]. Ganglionated plexi ablation is often added to thoracoscopic, video-assisted pulmonary vein antrum isolation [162]. Most studies investigating endocardial ganglionated plexi ablation have included paroxysmal AF patients but few, if any, persistent AF patients. Pokushalov *et al*. reported a low single procedure success rate of 38% after ganglionated plexi ablation without pulmonary vein isolation in longstanding persistent AF patients [163]. Currently, no study has randomized patients to CFAE ablation or ganglionated plexi ablation after pulmonary vein isolation. 

The exact mechanism by which CFAE ablation helps to improve arrhythmia-free outcome when performed during persistent AF ablation remains obscure and probably is a combination of different mechanisms. First, CFAE ablation might work because some important drivers of AF are ablated. As most CFAE sites are located close to high dominant frequency sites CFAE ablation may also isolate some of these high dominant frequency sites. Second, extensive left and right atrial CFAE ablation may help to decrease tissue anisotropy by micro-scar formation. As most CFAEs are a passive phenomenon occurring at complex anatomical sites, these sites will preferentially be ablated. Reduced anatomical complexity might downsize zones of slow conduction or zones prone to functional conduction block and reduce the number of re-entrant wavelets to the point where AF perpetuation is not possible anymore. Third, with wide circumferential pulmonary vein isolation ganglionated plexi or axons of the autonomic nerves may unintentionally be ablated, as these are located in the vicinity of the pulmonary vein antra [161]. 

### Summary

Persistent AF ablation involves substrate modification to achieve satisfying arrhythmia-free outcomes. Ablation of CFAE sites is the first add-on after pulmonary vein (antrum) isolation and effectively prolongs AF cycle length up to AF termination in a substantial proportion of patients. Definition of CFAEs is not uniform but usually should include both time and frequency domain. Most multicomponent CFAEs are a passive phenomenon and represent activation of adjacent structures or are the result of tissue anisotropy leading to fibrillatory conduction upon cycle length acceleration. Spectral analysis of atrial recordings are used to analyze the frequency domain of CFAEs. High dominant frequency sites may harbour important drivers of AF but do not necessarily overlap with regions of multicomponent CFAEs, although these can usually be found adjacent to high dominant frequency sites. CFAE sites demonstrating a greater percentage of continuous activity or a temporal activation gradient should be preferred ablation targets. Whether ablation of high dominant frequency sites translates into better outcome remains to be proven. Further studies are needed to better characterize CFAE sites critical for AF maintenance and which should preferentially be ablated to improve persistent AF ablation outcome.

## PERSISTENT AF ABLATION, CHALLENGE TWO: LINES

### Lines are a Mixed Blessing 

As discussed above, linear ablation is an important instrument in the electrophysiologist’s armamentarium against persistent AF. We favour to integrate linear lesions into a stepwise ablation approach aimed at AF termination and to use them specifically to target subsequent macro-reentrant atrial tachycardias. However, ablation of linear lesions is not without peril as achievement of complete linear block can be difficult or even impossible. Both incompletely ablated lines or recurred conduction across the line are associated with arrhythmia recurrence. Furthermore, verifying bidirectional conduction block across the line can be difficult and has some pitfalls. 

As soon as linear ablation is attempted, bidirectional conduction block across the ablated line should be the endpoint [164]. The majority of electrophysiology centres use conventional radiofrequency ablation for linear ablation and a non-steerable long sheath. For left atrial lines an irrigated, 3.5 mm tip catheter is preferred, while for right atrial lines non-irrigated, 8 mm tip catheters or other catheter types are also employed. The techniques on how to perform a roof line or mitral isthmus line and how to evaluate bidirectional block have been described in detail elsewhere [165-167]. Using radiofrequency ablation, the endpoint of bidirectional conduction block is not always achievable. After roof line ablation, complete linear block typically is observed in over 90% of patients [117, 122, 165]. The rate of complete linear block at the mitral isthmus line is somewhat lower at 66-92%, and in about two thirds of cases ablation from within the coronary sinus is required [117, 122, 166, 168-173]. 

**Fig. (6):**
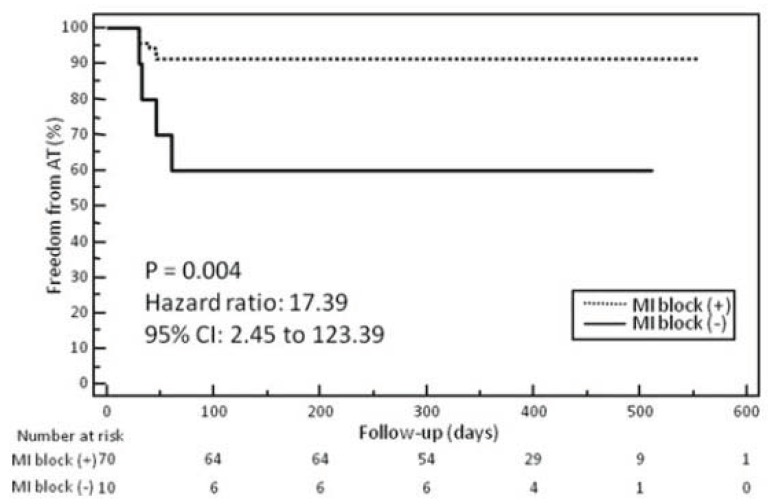
Kaplan–Meier curves of the incidence of atrial tachycardia after persistent AF ablation in patients with or without complete mitral isthmus (MI) block in the ablation procedure. Atrial tachycardia was significantly less frequently observed in patients who achieved mitral isthmus block compared to those who did not. Adapted from Matsuo *et al* [172].

A residual gap across an ablated line may have consequences. When complete linear block could not be achieved during the index procedure, the incidence of subsequent roof or mitral isthmus dependent macro re-entrant atrial tachycardias is higher (Fig. **6**) [122, 172]. But even with complete linear block during the index procedure conduction recovery across the ablated lines frequently occurs and is responsible for arrhythmia recurrence [118, 173-175]. For example, Pappone *et al*. randomized patients to circumferential pulmonary vein antrum ablation with or without additional posterior box lesion and mitral isthmus line [176]. Macro-reentrant atrial tachycardias were the most frequent type of recurrent atrial tachycardias in both groups, and a majority of them were related to gaps in ablated lines. Therefore, current technology and ablation techniques do not allow for effective and permanent linear lesions in a significant number of patients, and gaps in ablated lines are partly responsible for arrhythmia recurrence after persistent AF ablation using the stepwise ablation approach. 

### Factors Associated with Failed Linear Ablation

**Fig. (7):**
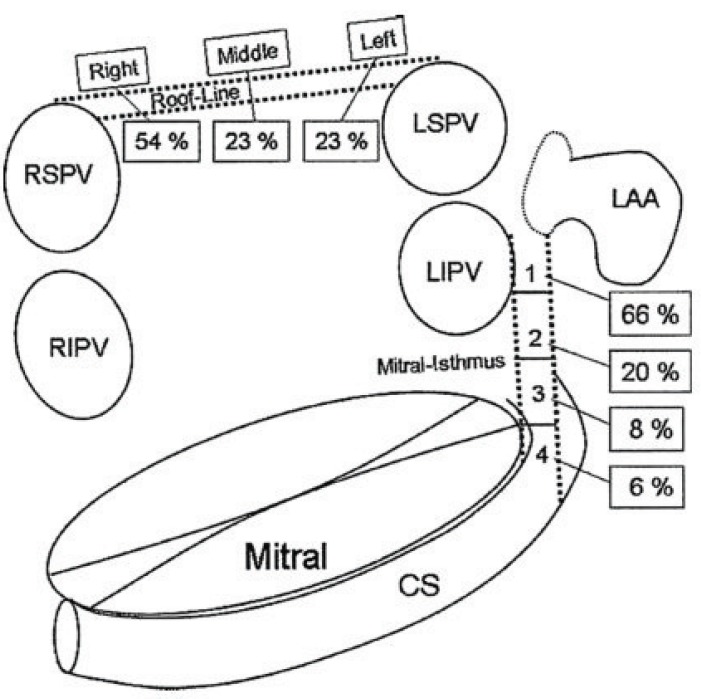
Schema of the left atrium and its adjacent structures. Dotted lines indicate linear lesions at the roof and mitral isthmus. The distribution of sites of conduction recovery at both the roof line and mitral isthmus is demonstrated. **CS**: coronary sinus; **LAA**: left atrial appendage; **LIPV**: left inferior pulmonary vein; **LSPV**: left superior pulmonary vein; **RIPV**: right inferior pulmonary vein; **RSPV**: right superior pulmonary vein. Adapted from Rostock *et al*. [177].

Many factors are responsible for the difficulties in achieving complete linear lesions and a high rate of conduction recovery across ablated lines. Rostock *et al*. analyzed gap locations across both the roof and mitral isthmus lines during repeat procedures in patients with recurrent arrhythmias after former linear ablations (Fig. **7**) [177]. Most gaps were located close to the pulmonary veins where catheter stability is often difficult to achieve: between the left inferior pulmonary vein and left atrial appendage for the mitral isthmus line and towards the right superior pulmonary vein for the roof line. 

Anatomical characteristics are a well described reason for ablation failure at the cavotricuspid isthmus [178]. The mitral isthmus also has a complex anatomical structure and several factors can oppose successful ablation. At the mitral isthmus line, both the great cardiac vein, as well as the circumflex artery, may act as a heat sink, preventing transmurality of ablated lesions. Kurotobi *et al*. analyzed the course of the circumflex artery in patients undergoing mitral isthmus ablation [179]. The rate of complete block at the mitral isthmus line was significantly lower in patients in whom the circumflex artery crossed the mitral isthmus line than in the remaining patients (42% versus 92%, p<0.001) and epicardial ablation was also more frequently necessary in these patients to obtain complete block (86% versus 27%, p<0.001). In the same study, the diameter of the great cardiac vein at the level of the mitral isthmus was inversely correlated with successful mitral isthmus ablation [179]. Overall, epicardial ablation from within the great cardiac vein is necessary in over two thirds of patients to achieve complete block at the mitral isthmus [166, 168, 170]. Apart from the great cardiac vein a prominent vein of Marshall can also act as a heat sink, limiting ablation efficacy at the junction between the left inferior pulmonary vein and the left atrial appendage [180]. In a case of a persistent left superior vena cava, ablation from within the abnormally dilated vein was necessary to achieve complete mitral isthmus block [181]. 

**Fig. (8):**
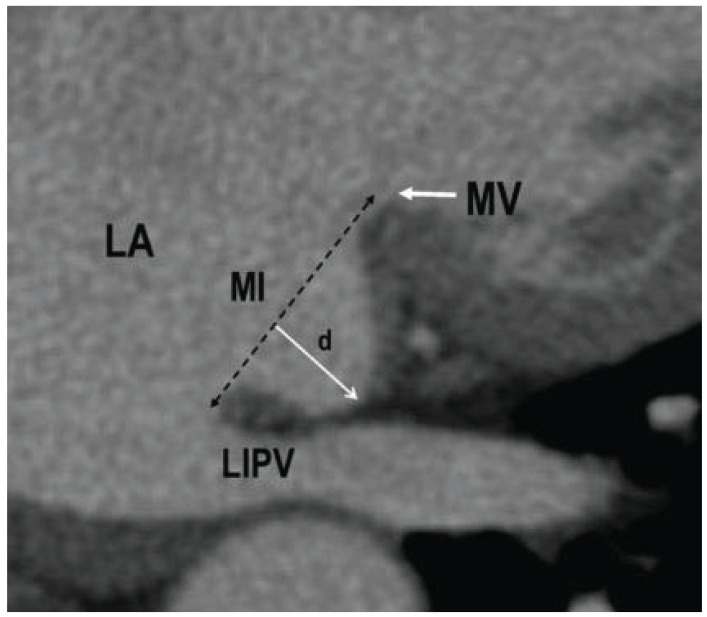
Example of a pouch at the mitral isthmus (MI). The depth (d) of the isthmus in this case was 14 mm. LA: left atrium; LIPV: left inferior pulmonary vein; MV: mitral valve. Adapted from Yokokawa *et al*. [170].

Computed tomography scans in 60 patients with persistent AF found a large range of left atrial wall thickness within in the left atrium, with the roof, left lateral ridge and mitral isthmus region having the thickest walls [182]. In computed tomography scans Yokokawa *et al*. looked at morphologic characteristics of the mitral isthmus that influence the acute efficacy of mitral isthmus ablation [170]. Patients with incomplete block were more likely to have a greater isthmus depth, a pouch at the isthmus (defined as isthmus depth >10 mm, (Fig. **8**)) and a higher prevalence of an interposed circumflex artery between the great cardiac vein and the mitral isthmus. Interestingly, wall thickness of the mitral isthmus was not correlated with ablation failure in that study. While the length of the mitral isthmus was inversely correlated with successful mitral isthmus ablation in the study by Kurotobi, this was not the case in the study by Yokokawa [170, 179]. In another study, patients with failed mitral isthmus ablation had a significantly higher take-off of the left inferior pulmonary vein and a trend for a longer mitral isthmus [171]. 

### New Techniques and Technologies for Permanent Linear Ablation

Two studies randomized patients undergoing mitral isthmus ablation to balloon-occlusion of the distal coronary sinus [183, 184]. Success rate in the balloon-occlusion group versus conventional group was not different in both studies. However, requirement of epicardial ablation was significantly reduced in both studies in the balloon-occlusion groups. This supports the hypothesis that the great cardiac vein acts as a heat sink which is why epicardial ablation is often necessary to obtain complete block. Miyazaki *et al*. performed left atrial linear ablations with a multielectrode catheter using duty-cycled bipolar and unipolar radiofrequency energy [185]. Complete block at the roof line was obtained in 60% of patients, and at the mitral isthmus line in 27% of patients only, a rate clearly inferior to conventional radiofrequency ablation when compared indirectly. Our group tested whether ablating a lateral mitral isthmus line or a posterior line results in a higher rate of complete block. We found no difference between the lines in term of success rate and total radiofrequency time (unpublished results). One of the major difficulties in both roof line and mitral isthmus line ablation is catheter stability [177]. Matsuo *et al*. randomized 80 patients to mitral isthmus ablation using a steerable sheath or a non-steerable sheath [172]. Rate of bidirectional mitral isthmus block was 98% in the group using a steerable sheath versus 78% in the other group (p=0.02) and epicardial ablation was needed less frequently when using the steerable sheath. As with pulmonary vein isolation, the integration of force feedback into catheter design might be advantageous for linear ablation as well. Force feedback may help the operator to improve catheter stability which could translate into better lesion control and success rate, but so far no studies have proved this. 

### Summary

Linear ablation is an essential component of the stepwise ablation approach. The aim of linear ablation is to compartmentalize the atrial substrate and to achieve a high rate of AF termination, which in turn is associated with better arrhythmia-free outcome. However, complete bidirectional block is often difficult to achieve, and both incompletely ablated lines or conduction recovery across ablated lines are responsible for recurrent arrhythmias. Anatomical reasons and inefficient ablation tools account for most ablation failures during linear ablation. Currently, only the use of a steerable sheath was able to increase acute ablation success for linear lesions at the mitral isthmus in a single study. Conduction recovery across ablated lines remains an inadequately addressed problem. Better lesion control with new catheters equipped with force-feedback function may help reduce conduction recovery, but so far no randomized studies have been performed. 

### General Conclusions and Perspectives

The reasons for failed AF ablation are different after paroxysmal and persistent AF ablation. Reconducting pulmonary veins are responsible for recurrent arrhythmia after paroxysmal AF ablation. Improvements in ablation technologies like force feedback and newer one-shot ablation devices are the most promising developments to reduce the rate of reconducting pulmonary veins. To increase success rate of persistent AF ablation, understanding of CFAEs critical for AF maintenance has to be advanced. Furthermore, tools allowing for safe, effective and durable linear ablation have to be developed. 

## Figures and Tables

**Table 1.** New Technologies for Pulmonary Vein Isolation

	Technology	Pros	Cons	Efficacy Compared to Conventional RF Ablation	Stage of Development

**Circular ablation catheters**					

PVAC®	Non-irrigated, circular multi-electrode RF ablation catheter	Circular ablation	No irrigation	Not inferior	Clinical use
		Duty-cycled bipolar/unipolar RF ablation	Silent cerebral ischemic lesions		
			Moderate pulmonary vein narrowing		
			Catheter diameter 9 Fr		
			Not adapted to all pulmonary vein anatomies		

nMARQ®	Irrigated, circular multi-electrode RF ablation catheter	Circular ablation	Catheter diameter 8.5 Fr	Results pending	Phase II trial
		Bipolar/unipolar RF ablation			
		External irrigation	Not adapted to all pulmonary vein anatomies		

**Balloon-based ablation catheters**					

Cryoballoon	Balloon-based ablation catheter delivering cryothermal energy	Safe	Phrenic nerve palsy	Not inferior	Clinical use
		Increased catheter stability during freezing	Catheter diameter 12 Fr		
			Not adapted to all pulmonary vein anatomies		
			Isolation of inferior veins technically more demanding		

HIFU balloon	Balloon-based ablation catheter with an integrated ultrasound cristal	Efficient lesion formation	High complication rate, including fatal outcome	Not compared	Abandoned

Thermal balloon	Balloon-based ablation catheter consisting of a saline-filled balloon heated by RF energy	Appears safe and effective	Catheter diameter 12 Fr	Not compared	Development stage
		Compliant balloon with variable diameter adapts to pulmonary vein anatomy			

Laser balloon	Balloon-based ablation catheter consisting of an endoscope and an arc generator laser delivery fibre	Visualization of the intravascular cardiac anatomy	More severe oesophageal thermal lesions reported	Not inferior	Clinical use
		Compliant balloon with variable diameter adapts to pulmonary vein anatomy	Catheter diameter 12 Fr		

**Pressure control**					

Steerable sheath	Conventional steerable sheath	Stability	Higher risk of perforation	Not inferior - superior	Clinical use
		Higher contact pressure			

Force feedback	RF ablation catheter capable of measuring real-time contact force	Pressure control	-	Results pending	Clinical use

**Remote navigation**					

Magnetic navigation	Magnetic navigation system allowing remote catheter manoeuvring	Improved catheter manoeuvrability and stability safe	Time consuming setup	Not inferior	Clinical use
			No tactile feedback (will be implemented in future)		

Robotic navigation	Electromechanical robotic system allowing remote catheter manoeuvring	Facilitated catheter manipulation	Sheath diameter 14 Fr	Not inferior	Clinical use
		Improved catheter contact and stability Tactile feedback	Vascular complications		

**Others**					

IRIS®ablation system	Endoscopic catheter allowing visualization of endocardial surface and irrigated RF ablation	Visualization of lesion formation and remaining gaps	Catheter diameter 12 Fr	Not compared	Development stage

Epicardial ablation	Minimally invasive, video-assisted, surgical epicardial ablation	Epicardial ablation	Higher adverse event rate	Comparison difficult	Clinical use
		Lesion visualization	Invasiveness		

RF: radiofrequency; HIFU: high-intensity focused ultrasound; PVAC: pulmonary vein ablation catheter

## ABBREVIATIONS

AF: = atrial fibrillation
CFAE: = complex fractionated atrial electrograms
